# Alterations of tumor suppressor gene *p16*^*INK4a *^in pancreatic ductal carcinoma

**DOI:** 10.1186/1471-230X-5-22

**Published:** 2005-06-28

**Authors:** Jyotika Attri, Radhika Srinivasan, Siddhartha Majumdar, Bishan Dass Radotra, Jaidev Wig

**Affiliations:** 1 Department of General Surgery, Postgraduate Institute of Medical Education and Research, Chandigarh, India; 2 Department of Cytology and Gynaec Pathology, Postgraduate Institute of Medical Education and Research, Chandigarh, India; 3 Department of Experimental Medicine and Biotechnology, Postgraduate Institute of Medical Education and Research, Chandigarh, India; 4 Department of Histopathology, Postgraduate Institute of Medical Education and Research, Chandigarh, India

## Abstract

**Background:**

Cell cycle inhibitor and tumor suppressor gene *p16 / MTS-1 *has been reported to be altered in a variety of human tumors. The purpose of the study was to evaluate primary pancreatic ductal adenocarcinomas for potentially inactivating *p16 *alterations.

**Methods:**

We investigated the status of *p16 *gene by polymerase chain reaction (PCR), nonradioisotopic single strand conformation polymorphism (SSCP), DNA sequencing and hypermethylation analysis in 25 primary resected ductal adenocarcinomas. In addition, we investigated p16 protein expression in these cases by immunohistochemistry (IHC) using a monoclonal antibody clone (MS-887-PO).

**Results:**

Out of the 25 samples analyzed and compared to normal pancreatic control tissues, the overall frequency of *p16 *alterations was 80% (20/25). Aberrant promoter methylation was the most common mechanism of gene inactivation present in 52% (13/25) cases, followed by coding sequence mutations in 16% (4/25) cases and presumably homozygous deletion in 12% (3/25) cases. These genetic alterations correlated well with *p16 *protein expression as complete loss of p16 protein was found in 18 of 25 tumors (72%).

**Conclusion:**

These findings confirm that loss of p16 function could be involved in pancreatic cancer and may explain at least in part the aggressive behaviour of this tumor type.

## Background

Pancreatic cancer is a malignant neoplasm in the digestive tract the etiology of which is not known fully as yet. Recent advances in molecular oncology have provided explanations at the DNA level that multiple genetic changes contribute to pancreatic cancer development in which the *p16 *locus of tumor tissue is nearly always altered [1]. The *p16 *tumor suppressor gene located on 9p21 enodes a 16 KDa protein that acts as a cyclin dependent kinase (CDK) 4/6 inhibitor [2].

p16 belongs to an important group of proteins that includes the p15^INK4b^, p21^waf1 ^and p27^KIP1 ^which negatively regulate the G_1 _phase of cell cycle [2]. The *p16 *gene product binds to CDK4 and CDK6 inhibiting their interaction with cyclin D1. The inhibition of cyclin D1-CDK4/6 complex activity prevents retinoblastoma protein phosphorylation and release of E2F, leading to the inhibition of cell cycle in G1/S transition [3]. Genetic abnormalities inactivating the *p16 *gene thus confer a growth advantage to the cell contributing to tumorigenesis.

Inactivating alterations of the gene have been commonly identified in a number of human malignancies [4,5]. In cancers, functional loss of *p16*^*INK4a *^occurs as a consequence of somatic mutations, homozygous and heterozygous deletions [6,7]. A high frequency of homozygous deletion and mutation of this gene have been detected in cell lines derived from different types of tumors (glioma, breast, lung, bladder and melanoma) [6,8] suggesting that *p16 *may play an important role in the regulation of cellular growth in the majority of cell types. However, homozygous deletions and somatic mutations are rarely observed in primary tumors with *p16 *genetic alterations [9,10]. On the other hand, denovo methylation has also been proposed to be an important alternative mechanism of *p16 *gene inactivation [11]. DNA hypermethylation can inhibit transcription of tumor suppressor genes and mismatch repair genes (*p16, hMLH1 and VHL*), providing an epigenetic mechanism of selection during tumorigenesis [11,12].

Abnormalities of tumor suppressor gene *p16 *have been reported in a variety of human tumors but less information is available regarding alterations of *p16 *in primary pancreatic ductal carcinoma than in pancreatic cancer derived cell lines and xenografts. There have been a few reports on the *p16 *alterations in tissue specimen of primary pancreatic ductal adenocarcinomas till date [13-17]. To provide further evidence that the cell cycle inhibitor *p16 *might be relevant for pancreatic tumorigenesis, we performed a comprehensive analysis of the mechanisms involved in *p16 *inactivation such as mutation, hypermethylation and homozygous deletion, in primary ductal adenocarcinomas.

## Methods

### Tissue samples

A written informed consent was obtained from each patient for inclusion in this study which was carried out after obtaining a formal approval from the Institute Ethics Committee. Pancreatic cancer tissues were obtained from 25 patients (15 males, 10 females) undergoing surgery for pancreatic cancer. Normal tissue away from the main tumor mass was taken as control. The age of these patients ranged from 27–78 years. According to the tumor, node, metastases classification of International Union against cancer [18], there were 2 patients with stage I, 8 patients with stage II, 14 patients with stage III and one patient with stage IV disease.

Freshly removed pancreatic tissue samples were immediately fixed in 10% formalin for 24 hours and paraffin embedded. A section of each specimen was stained with hematoxylin and eosin and microscopically examined to confirm the diagnosis. In addition, fresh tissues for molecular analysis were frozen and stored at -80°C until use. One frozen section from each tissue subjected to molecular analysis was assessed histologically to ensure the presence of tumor and only those samples which contained >90% of tumor were included in the final analysis.

### Detection of homozygous deletion in *p16*^*INK4a*^

Genomic DNA was isolated from the stored frozen tissue using the parallel RNA/DNA extraction kit (Qiagen, GmbH, Germany). PCR was performed using 100 ng template DNA and 10 pmol of each primer (Biobasic INC, Canada) in a volume of 50 μl containing 10 mM Tris HCl (pH 8.3), 50 mM KCl, 1.5 mM MgCl_2_, 2.0 mM of each dNTPs and 1.5 units *Taq *polymerase (ROCHE, GmbH, Germany). The following primer pairs were used:

Exon 1: 5' cggagagggggagaacagac 3' and 5' ctggatcggcctccgaccgtaac 3' 189 bp

Exon 2: 5' tgagggaccttccgcggc 3' and 5' gtcatgatgatgggcagcgc 3' 307 bp

Exon 3: 5' cacatccccgattgaaagaac 3' and 5' cagtgaatgaatgaaaatta 3' 489 bp

These primers were designed by us based on their mRNA sequence available in the Genbank [Genbank: 4502748]. The PCR program was set for an initial denaturation at 95°C for 5 min, 35 cycles of denaturation at 95°C for 1 min, annealing at 55–56°C (depending on primer pair), for 1 min, extension at 72°C for 1 min and final extension at 72°C for 7 min. PCR reaction products were electrophoresed on a 1.5% agarose gel and were visualized under UV illuminator. Each time, a positive control (normal lymphocyte DNA) was included in the PCR reaction.

### Detection of mutation in *p16*^*INK4a *^gene by single strand conformation polymorphism (SSCP) analysis

All samples were analysed for mutation in exon 1, 2 and 3 of *p16 *by SSCP [19] and DNA sequencing of the PCR products revealing a mobility shift.

For SSCP analysis, 1 μl of each PCR product formed using the above mentioned PCR program and primers, was mixed with 9 μl SSCP loading buffer (98% v/v, formamide, 10 mM EDTA+0.05% Bromo-Phenol Blue+0.5% Xylene Cyanol) and incubated at 90°C for 5', followed by rapid cooling on ice. 10 μl of this mixture was loaded on a 6% acrylamide gel containing 1XTrisborate EDTA buffer. Following electrophoresis at 50 V at 4°C for 15–20 hrs, the gel was analysed by silver staining [20].

The presence of bands with variant migration pattern was confirmed by repeating PCR-SSCP at least prior to extraction of band for DNA sequence analysis.

### DNA sequencing

PCR products that revealed mobility shift on SSCP analysis were send for DNA sequencing. Automated sequencing reactions were carried out using Perkin Elmer Big Dye sequence Terminator Mix (ABI / PE, Foster, CA) as per manufacturer's instructions and sequenced on an ABI 377 sequencer.

### Methylation analysis of *p16*^*INK4a *^gene

The methylation status of 5' CpG islands of *p16 *gene by bisulfite modification of DNA and Methylation Specific PCR (MS-PCR) was performed according to the method of Herman et al [21].

Briefly, DNA (1 μg) in a volume of 50 μl was denatured by 0.2 M NaOH for 10' at 37°C, then 30 μl of 10 mM hydroquinone (Sigma, St. Louis, USA) & 520 μl of 3 M Na bisulfite, pH 5.0 (Sigma, St. Louis, USA), both freshly prepared were added to each sample. These were then incubated at 50°C/16 h. Modified DNA was purified using the wizard DNA purification Kit (Promega, Madison, WI, USA). Modification was completed by 0.3 M NaOH treatment for 5 min at room temperature, followed by ethanol precipitation, DNA was resuspended in distilled water and stored at -20°C.

### PCR amplification

Sequences of primer pair (Biobasic, INC, Canada) used for MSPCR on *p16*^*INK4a *^gene were: unmodified or wild type primers (W), 5' cagaggtggggcggaccgc 3' and 5' cgggccgcggccgtgg 3'; methylated specific primers (M), 5' ttattagagggtggggcggatcgc 3' and 5' gaccccgaaccgcgaccgtaa 3'; unmethylated specific primers (U) 5' ttattagagggtggggtggattgt 3' and 5' caaccccaaaccacaaccataa 3'. PCR products identified by W, M and U primers were 140 bp, 150 bp and 151 bp respectively.

The PCR mixture contained 1 × PCR buffer (16.6 mM ammonium sulphate / 67 mM Tris, pH 8.8 / 6.7 mM MgCl_2 _/ 10 mM 2-mercaptoethanol) dNTPs (each at 2 mM), Primers (100 pmol) and bisulfite treated DNA (~50 ng) or unmodified DNA (50–100 ng) in a final volume of 50 μl. PCR specific for unmodified DNA also included 5% DMSO. Reactions were hot started 95°C for 5' before the addition of 1.5 units of *Taq *polymerase (ROCHE, GmbH, Germany).

Amplification was carried out in the following conditions, 35 cycles at 95°C (30 sec), 60–65°C (depending on the type of primer pair used) (30 sec), 72°C (30 sec) followed by a final 5 min extension at 72°C. Each PCR product was loaded onto a 2% agarose gel, stained with ethidium bromide and visualized under UV illuminator. DNA from Raji cell line was used as a positive control for methylated alleles of this gene. DNA from normal lymphocyte was used as the control for unmethylated alleles.

### Immunohistochemistry

Immunohistochemical detection was performed according to the avidin biotin complex method using the ABC staining kit (Santa Cruz Biotech INC., CA, USA). In brief, 5 μm sections were cut from formalin fixed, paraffin embedded tissue specimens. After treatment with blocking solution (0.03% H_2_O_2 _in methanol) to block endogenous peroxidase activity, the antigenic sites were unmasked by means of 3 cycles of 5 minutes microwave irradiation in 10 mM citrate buffer (pH 6.0). Sections were then incubated with the primary antibodies (Neomarkers, Fremont, USA) against p16 (clone MS-887-PO) used at 1:20 dilution, for 2 hours at room temperature. Sections were further incubated with the secondary biotinylated antibody followed by treatment with ABC reagent. The slides were developed using 3-3' diaminobenzidine as the chromogen and counterstained with hematoxylin followed by mounting with DPX.

The IHC results were scored by taking percentage positivity and intensity of staining into account. An intensity score of 0 = No staining, 1 = weak positivity; 2 = moderate positivity & 3 = strong positivity was given.

### Statistical analysis

The following statistical tests were applied to analyze the data:

Wilcoxon Signed Rank Test, Chi Square Test and Pearson Correlation Coefficient Test. A probability value of less than 0.05 was considered to be significant.

## Results

Paired normal and tumor DNA from 25 patients with pancreatic cancer were examined for the occurrence of *p16 *genetic alterations.

### Homozygous deletion

**Figure 1:**
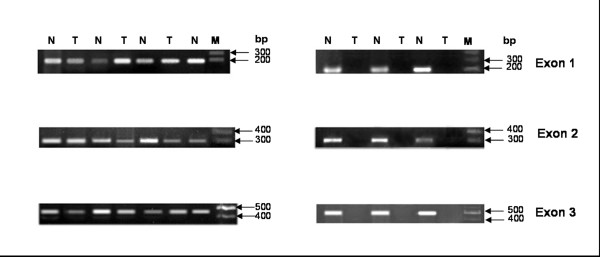
PCR analysis of *p16 *exon 1, 2 & 3 in normal pancreas and pancreatic tumor. M: Molecular weight marker; N: Normal Pancreas; T: Pancreatic Cancer.

We separately amplified exon 1, 2 & 3 of *p16 *using specific primers for homozygous deletion and for SSCP analysis of PCR products. In 3 out of 25 (12%) tumors, we failed to amplify exon 1, 2 and 3 whereas these three exons could be amplified from the corresponding normal pancreatic tissue (Fig 1). Hence, in these three cases there was presumably a homozygous deletion of the *p16 *gene.

### SSCP analysis

**Figure 2:**
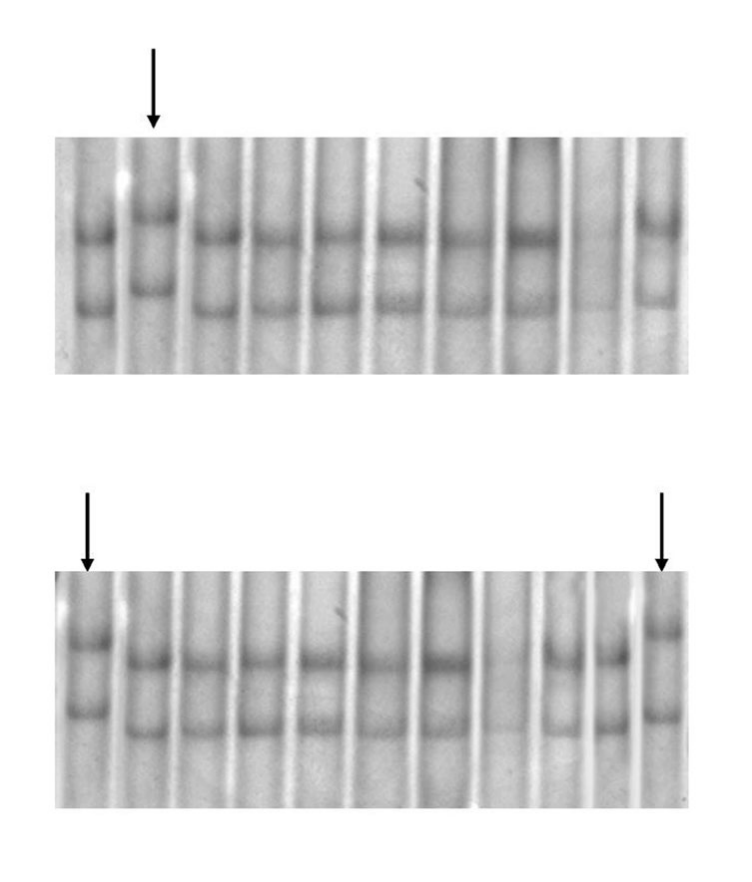
PCR-SSCP analysis of *p16 *mutations in pancreatic cancer samples. Arrows indicates bands with a mobility shift.

**Figure 3:**
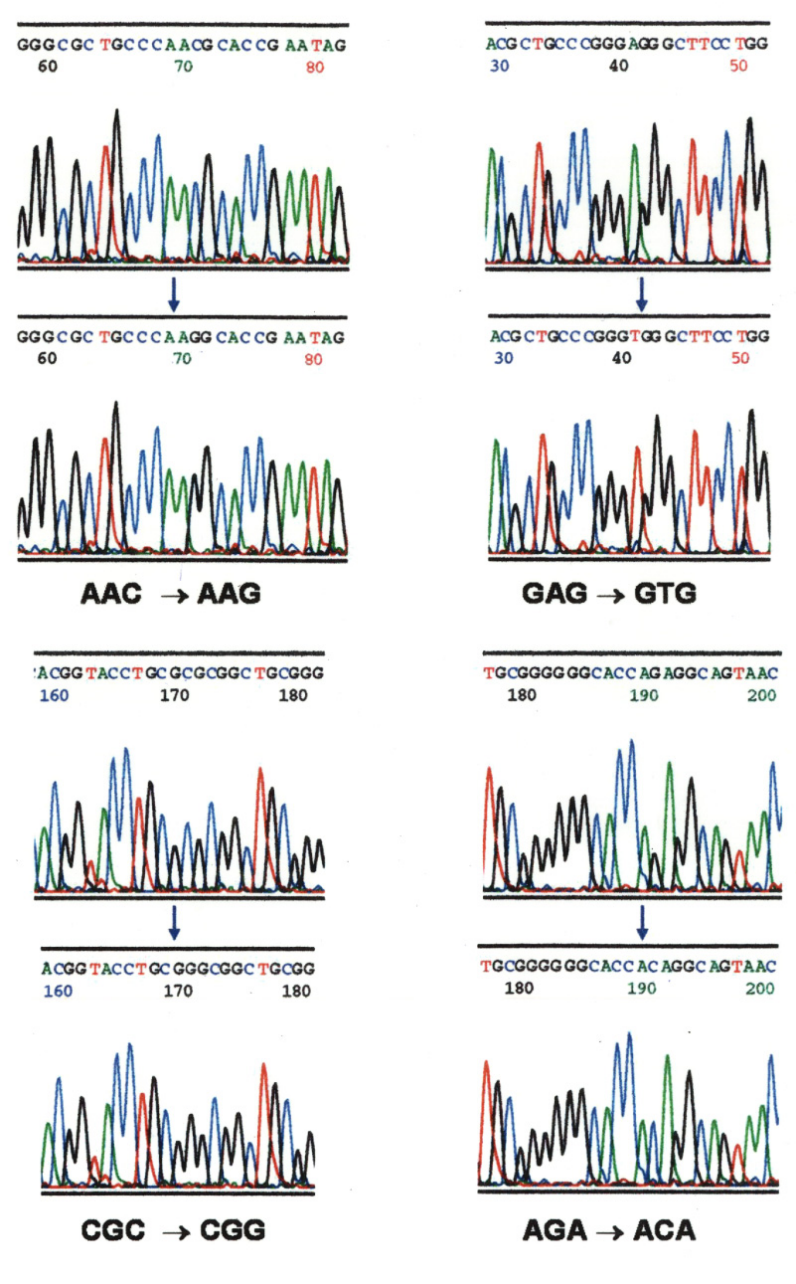
Automated DNA sequencing results to determine point mutations in *p16 *exon 1 and 2.

To screen for mutation in *p16 *gene, exon 1, 2 and 3 were analysed by PCR-SSCP. 5 out of 25 cases analysed showed evidence for *p16 *mutation (exon1, one case; exon 2, four cases) (Fig 2). No mobility shift of exon-3 was found in any sample. DNA samples showing electrophoretic band shift mobility were reamplified and the product purified and used directly for sequencing. DNA sequence analysis of these abnormally migrating SSCP fragment revealed point mutations in 4 out of 5 cases (3 transversions and 1 missense mutation) (Fig. 3). Table 1 summarizes the SSCP and DNA sequencing data for these.

### Methylation analysis of *p16*

**Figure 4:**
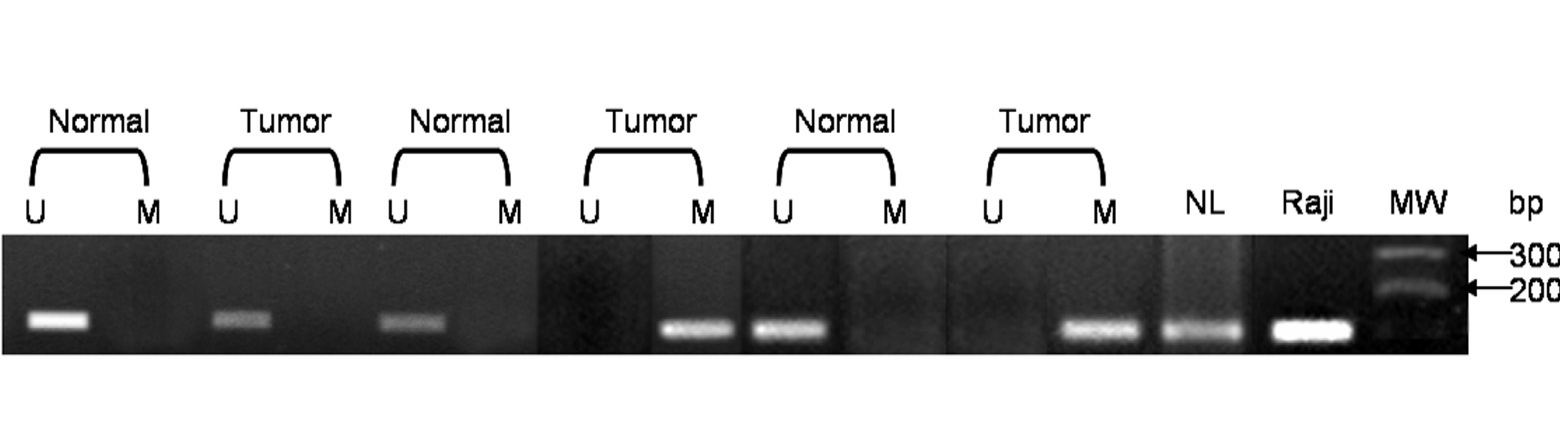
Methylation specific PCR of *p16 *gene in normal pancreas and in pancreatic cancer. M: PCR with primers for methylated *p16*. U: PCR with primers for unmethylated *p16*. NL : Normal Lymphocyte DNA used as a negative control for methylation. Raji: Cell line DNA used as a positive control for methylation. MW: Molecular weight marker.

Methylation status of *p16*^*INK4a *^gene was evaluated in 25 tumors. A total of 13 (52%) samples showed evidence of promoter methylation. In 11 out of these 13 cases, the gene was completely methylated while in the other 2 cases the gene was partially methylated (Fig. 4).

### p16 immunohistochemistry

**Figure 5:**
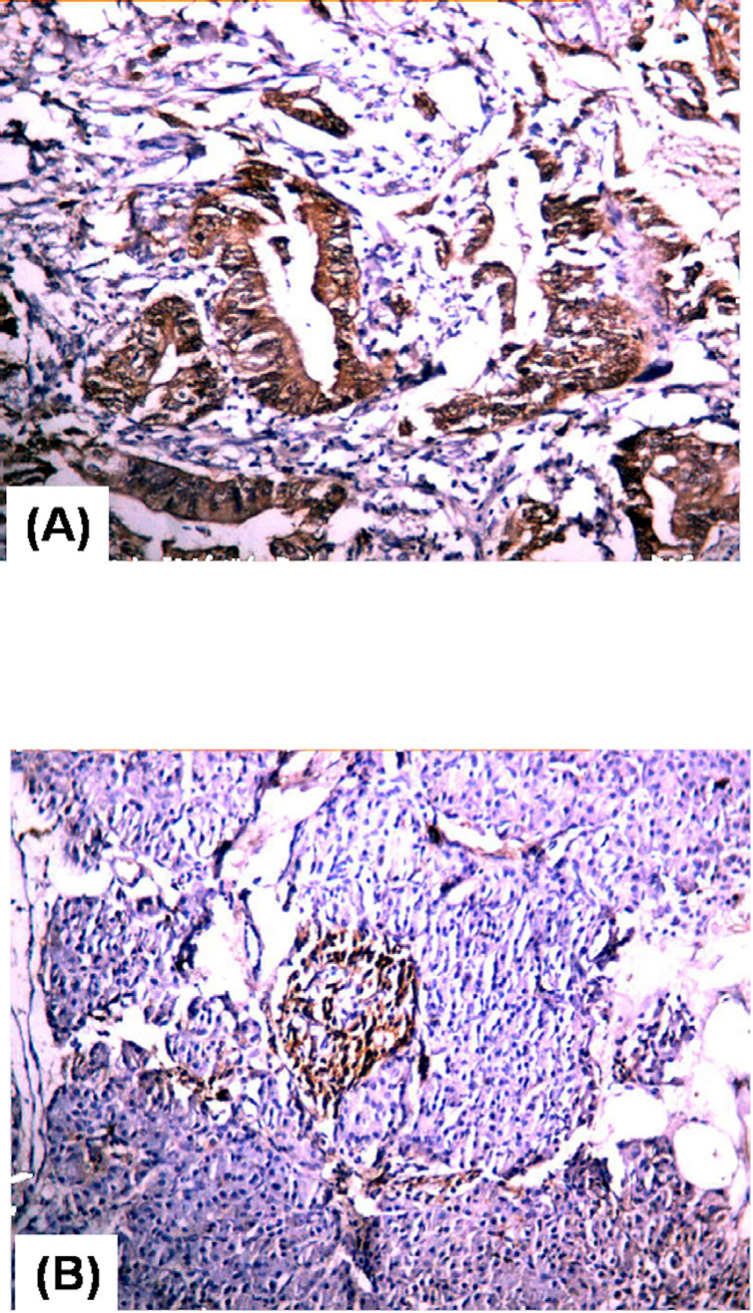
Immunohistochemical staining of p16 in representative cases of (A) pancreatic cancer (B) Normal pancreas (original magnification ×200).

Loss of p16 protein expression was noted in 18 out of 25 tumors as determined by immunohistochemistry. However, 7 out of 25 (28%) ductal adenocarcinomas stained positive for p16 with weak/focal p16 nuclear staining in 4 and moderate positivity in 3 cases (Fig. 5A). In normal pancreas, p16 nuclear positivity was noted in islets of Langerhans with scattered non-specific cytoplasmic positivity in ductal and acinar cells (Fig. 5B).

### Comparison of *p16 *gene alterations to p16 protein expression

The *p16 *gene alterations were compared to the p16 protein expression and the results are tabulated in Table 2. In the 18 adenocarcinomas negative for p16 expression, 11 had methylation of the promoter region. The *p16 *gene was deleted in 3 cases with mutations detected in 2 additional cases. However, two tumors negative for p16 expression did not reveal any of the genetic alterations.

### Statistical analysis

These genetic alterations showed a significant correlation with the p16 protein expression (Pearson Correlation Coefficient Test and Chi Square Test, p < 0.01). However, no correlation was found between *p16 *gene alterations (mutation, deletion and hypermethylation) and age, TNM staging and histological differentiation (Wilcoxon Signed Rank Test and Chi Square Test, p > 0.05).

## Discussion

The *Rb / p16 *tumor suppressive pathway can be abrogated in tumors by inactivation of any of the several members of the pathway [22,23]. For pancreatic carcinoma, this disruption is caused exclusively by inactivation of *p16*^*INK4a *^gene and, only rarely, the *Rb *gene [24,25]. Inactivation of *p16 *gene occurs through intragenic mutation, homozygous deletion and methylation associated transcriptional silencing. In the present study, all known mechanisms of *p16 *inactivation were analysed and we found evidence of *p16 *inactivation in a high proportion (80%) of the pancreatic tumors examined.

Most of the previous studies regarding pancreatic cancer have reported an elevated frequency of *p16 *gene alterations in pancreatic cancer derived cell line and xenografts [7,13,26,27]. There have been a few reports on the *p16 *alterations in tissue specimen of primary pancreatic ductal adenocarcinomas. Our report is the sixth such study and the first from India. A comparison of the result of all the reports with the present study is shown in Table 3. Homozygous deletion could not be detected in two studies by Gerdes et al [14] and Ohtsubo et al [15], but were observed in 35% cases in the study by Zhonghua et al [16]. In our study, we were unable to amplify the *p16 *exons 1–3 in 12% cases presumably indicating homozygous deletion which is in good agreement with 10% deletion frequency reported by Huang et al [13]. We identified mutation in 4 out of 25 (16%) cases which is consistent with 15% mutation frequency reported by Ohtsubo et al [15]. However, this frequency is lower than 22.5% reported by Gerdes et al [14]. Also, the frequency of mutation was higher in exon 2 than exon 1 (3 cases in exon 2 compared with 1 case for exon 1). This result is consistent with the previous reports [13,14]. Of the 4 points mutations reported here, 3 were transversions and 1 was a missense mutation. Importantly, one out of these four mutations (glutamine → valine at codon 88) was located in the ankyrin repeat III in a highly conserved region believed to form the CDK binding cleft and may have resulted in loss of *p16 *function [28]. Out of the remaining three mutations, one was in ankyrin repeat I and two others were in ankyrin repeat IV. None of these mutations involved the conserved amino acid and hence may not be crucial to p16 function [28]. On the other hand, two of these mutations resulted in a loss of p16 protein expression and so, it may be postulated that they could have affected the stability and half life of the mRNA or protein. This postulation needs confirmation by further experimental approaches. In 2 out of 18 tumors negative for p16 protein expression, we were unable to detect any genetic alteration in the form of deletion, mutation or promoter hypermethylation. However, presence of a mutation is not completely excluded, as we used PCR-SSCP as a screening tool and it is known from the published studies that this technique detects only about 70% of mutations [19,29].

We have observed hypermethylation to be a major mechanism of *p16 *inactivation. This frequency is higher than the 27% frequency reported by Gerdes et al [14] and Fukushima et al [30]. Other studies report and even lower frequency of 15% and 3.3% [15,17]. The later study was however performed on microdissected tissue samples using restriction enzyme analysis in contrast to our study on fresh samples using MSPCR. Another explanation for the observed differences in the methylation frequencies could be differences in the ethnicity of the population studied and the role of unknown environmental factors. Such a difference in the methylation profile is reported in the context of hepatocellular carcinomas by Shen et al [31]. In pancreatic cancer cell lines also, the prevalence of *p16 *promoter methylation has ranged from 18%-38% [27,32]. *p16 *promoter methylation has also been detected in pancreatic intraductal neoplasia adjacent to pancreatic cancer [28,33,34]. Moreover, the detection of *p16 *promoter methylation in the pancreatic fluid of patients with pancreatic cancer but not in chronic pancreatitis patients has suggested its potential role as a diagnostic marker in the differentiation of benign and malignant pancreatic disease [35].

Most of the previous studies which have correlated *p16 *alterations to survival data are limited by the fact that a comprehensive analysis of the *p16 *genetic alterations is lacking. Bartsch et al [36] found reduced survival in patients with *p16 *mutations but this study did include the analysis of *p16 *inactivation by homozygous deletions. Hu et al [37], found longer survival in patients with immunohistochemically p16 positive tumors compared with p16-negative ones, but this difference was not significant. Naka et al [38], identified a significantly longer survival in IHC p16 positive tumors, but the analysis of gene mutations was not examined in these studies. The association of *p16 *alterations with worse prognosis has also been reported by Gerdes et al [14] and Ohtsubo et al [15]. In the present analysis, although correlation to survival was not possible, *p16 *alterations did not show any association with the stage or histological grade of the tumor.

## Conclusion

Overall, our findings support the observations that *p16 */ MTS-1 gene alterations play a key role in dysregulated growth of pancreatic cancer and that methylation of the promoter is an important mechanism of its inactivation in Indian patients with pancreatic ductal adenocarcinoma.

## Competing interests

The author(s) declare that they have no competing interests.

## Authors' contributions

SJ carried out the molecular genetic studies and drafted the manuscript. SR participated in its design, coordination and helped to draft the manuscript. MS did DNA sequencing part of the study and coordinated the study. DRB did immunohistochemical part of the study. WJ provided the tissue samples and all the clinical information regarding the patient. All authors have read and approved the final manuscript.

## Pre-publication history

The pre-publication history for this paper can be accessed here:



## Figures and Tables

**Table 1:** *p16 *sequence changes in pancreatic cancer.

**Sample**	**Exon**	**Codon**	**Nucleotide Altered**	**Predicted Product**
S-4	1	39	AA**C **→ AA**G**	Asparagine → Lysine
S-10	2	132	CG**C **→ CG**G**	Arginine → Arginine
S-17	2	88	G**A**G → G**T**G	Glutamine → Valine
S-22	2	139	A**G**A → A**C**A	Arginine → Threonine

**Table 2:** Comparison of p16 protein expression to the *p16 *gene alterations.

**Sr. No.**	**p16 protein expression**	***p16 *gene alteration**		
		
		**Deletion**	**Mutation**	***p16 *promoter methylation**
1.	-	-	-	+ / -
2.	-	-	-	+ / -
3.	-	+	-	- / -
4.	-	-	+	- / +
5.	+	-	-	+ / +
6.	-	-	-	+ / -
7.	-	+	-	- / -
8.	-	-	-	+ / -
9.	-	-	-	+ / -
10.	+	-	+	- / +
11.	-	-	-	+ / -
12.	-	+	-	- / -
13.	-	-	-	+ / -
14.	+	-	-	+ / +
15.	-	-	-	+ / -
16.	-	-	-	- / +
17.	-	-	+	- / +
18.	-	-	-	+ / -
19.	+	-	-	- / +
20.	+	-	-	- / +
21.	-	-		+ / -
22.	+	-	+	- / +
23.	-	-	-	+ / -
24.	+	-	-	- / +
25.	-	-	-	- / +

+ / + : Completely methylated

- / - : Unmethylated

+ / - : Partially methylated

**Table 3:** Studies showing *p16 *alterations in tissue specimen of primary pancreatic ductal adenocarcinomas.

**Study**	**Number of cases**	**p16 gene alterations**		
		
		**Homozygous deletion**	**Mutation**	**Methylation**
Huang et al (1996)	30	10%	17%	NE
Zhonghua et al (2000)	35	35%	20%	NE
Moore et al (2002)	34	NE	23%	15%
Gerdes et al (2002)	40	ND	22.5%	27.5%
Ohtsubo et al (2003)	60	ND	15%	3.3%
Present study	25	12%	16%	52%

NE: Not Examined; ND: Not Detected
